# H.O.S.T.: Hemoglobin microbubble-based Oxidative stress Sensing Technology

**DOI:** 10.1038/s41598-023-42050-z

**Published:** 2023-09-11

**Authors:** Antra Ganguly, Sugandha Chaudhary, Shashank R. Sirsi, Shalini Prasad

**Affiliations:** Department of Bioengineering, The University of Texas at Dallas, Richardson, TX 75080 USA

**Keywords:** Sensors and probes, Diagnostic markers, Cancer screening, Biomedical engineering

## Abstract

In this work, we discuss the development of H.O.S.T., a novel hemoglobin microbubble-based electrochemical biosensor for label-free detection of Hydrogen peroxide (H_2_O_2_) towards oxidative stress and cancer diagnostic applications. The novelty of the constructed sensor lies in the use of a sonochemically prepared hemoglobin microbubble capture probe, which allowed for an extended dynamic range, lower detection limit, and enhanced resolution compared to the native hemoglobin based H_2_O_2_ biosensors. The size of the prepared particles Hemoglobin microbubbles was characterized using Coulter Counter analysis and was found to be 4.4 microns, and the morphology of these spherical microbubbles was shown using Brightfield microscopy. The binding chemistry of the sensor stack elements of HbMbs’ and P.A.N.H.S. crosslinker was characterized using Attenuated Total Reflectance Fourier Transform Infrared Spectroscopy and UV–Vis Spectroscopy. The electrochemical biosensor calibration (R^2^ > 0.95) was done using Electrochemical Impedance Spectroscopy, Cyclic Voltammetry, and Square Wave Voltammetry. The electrochemical biosensor calibration (R^2^ > 0.95) was done using Electrochemical Impedance Spectroscopy, Cyclic Voltammetry, and Square Wave Voltammetry. The specificity of the sensor for H_2_O_2_ was analyzed using cross-reactivity studies using ascorbic acid and glucose as interferents (*p* < 0.0001 for the highest non-specific dose versus the lowest specific dose). The developed sensor showed good agreement in performance with a commercially available kit for H_2_O_2_ detection using Bland Altman Analysis (mean bias = 0.37 for E.I.S. and − 24.26 for CV). The diagnostic potential of the biosensor was further tested in cancerous (N.G.P.) and non-cancerous (H.E.K.) cell lysate for H_2_O_2_ detection (*p* = 0.0064 for E.I.S. and *p* = 0.0062 for CV). The Michaelis Menten constant calculated from the linear portion of the sensor was found to be ${K}_{m}^{app}$ of 19.44 µM indicating that our biosensor has a higher affinity to Hydrogen peroxide than other available enzymatic sensors, it is attributed to the unique design of the hemoglobin polymers in microbubble.

## Introduction

Hydrogen peroxide is the most investigated R.O.S. as it is the most stable reactive oxygen species^1^. It is an essential metabolite in cellular redox metabolism reactions and processes. It is a critical metabolite for most cells’ redox metabolism reactions and processes. It is well known that cancer cells are under oxidative stress than normal cells due to higher levels of Reactive Oxygen Species^2^. The R.O.S. is also responsible for tumor transformation^3^, progress^4^ and survival^5^. Therefore, monitoring oxidative stress in cancer cells is of utmost importance. The standard methods of hydrogen peroxide detection are colorimetry^6^, chemiluminescence^7^, and electrochemistry^7^. Optical sensing methods like chemiluminescence suffer from interference from other light sources and require complex instrumentation, whereas colorimetry shows false positives and is ineffective in turbid samples^8^**.** Electrochemical techniques, on the other hand, are widely used because of their ability to miniaturize sensors, excellent sensitivity, and reproducibility^9^. Hemoglobin’s intrinsic peroxidase-like activity makes it an appropriate substitute for other similar enzymes, such as horseradish peroxidase, specifically for improving the biosensor performance and reducing the fabrication cost. Although hemoglobin (Hb) contains four redox-active iron centers, direct detection is not possible due to the inaccessibility of the iron center and the formation of dimers, blocking electron transfer^10,11^.

Current solutions involve many strategies that improve the immobilization technique of Hemoglobin onto electrodes and better charge transfer to the electrode, such as metal–organic frameworks^12^, which are used to encapsulate hemoglobin and carbon nanotube and mesoporous silica particles to absorb hemoglobin for biosensing applications^13^ and covalent immobilization of hemoglobin^14^. These strategies discuss improving the catalytic activity of the hemoglobin but do not discuss the conformational mobility of the hemoglobin protein. In the most recent study done using Hydrophobic ionic liquid @ hemoglobin, which was synthesized using microfluidic technology^15^, the main drawback of this Technology is that it is costly for proposed particle synthesis, making it inaccessible for resource-limited areas. Apart from the biosensors discussed above, supplementary Table 1 shows the biosensors for H_2_O_2_ detection currently available in the research space.

**Figure 1:**
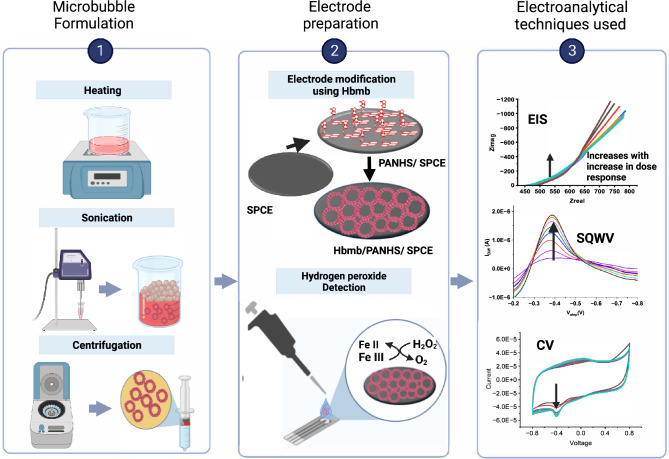
Schematic of the hemoglobin microbubble formation, the electrode modification using the hemoglobin microbubble and the three electroanalytical methods used to show the Hydrogen peroxide sensing ability of the modified electrode.

Through this work, we propose the development of a novel gas (in this case air) filled hemoglobin microbubble-based electrochemical biosensing platform, H.O.S.T (Hemoglobin Microbubble based Oxidative Stress Sensing Technology), for highly sensitive and specific detection of H_2_O_2_ in biological samples supplementary Figure S6) shows hydrogen peroxide release in cancer cells) By loading high amount of hemoglobin in the microsphere^16^ using sonochemical method^17^ (Fig. 1).

At its core, H.O.S.T. is an affinity biosensor that leverages the redox chemistry between the heme sites of the microbubble capture probe and the hydrogen peroxide species expressed in the sample of interest developed sensor shows a significant enhancement in the sensor metrics compared to a simple native hemoglobin-modified electrode for hydrogen peroxide detection. Hemoglobin microbubbles were immobilized on the carbon working electrode (of a standard 3-electrode system) using a P.A.N.H.S. crosslinker via π–π interaction, validated using Attenuated Total Reflectance Infrared Spectroscopy, UV–Vis Spectroscopy techniques confirmed that the hemoglobin protein maintained its conformity throughout the production and crosslinking process. UV–Vis Spectroscopy techniques. The interaction of the capture probe with the target H_2_O_2_ molecules led to changes in dielectric properties and the rearrangement of water molecules and ionic species at the electrode–electrolyte interface, which were captured using a combination of an established Cyclic Voltammetry, Square wave Voltammetry, and Electrochemical Impedance Spectroscopy for a wide dynamic range in PBS. The performance and diagnostic efficacy were tested using real cell lysate samples, and the sensor could reliably distinguish between the H_2_O_2_ levels in cancerous vs. non-cancerous cells with high statistical significance using both E.I.S. and C.V. The accuracy of sensor performance was validated against a commercially available absorbance-based hydrogen peroxide detection kit. This is the first reported study to use Hemoglobin microbubble on screen-printed carbon electrode without any other modification for hydrogen peroxide quantification. We characterized the sensor's working using 2 D.C. and 1 A.C.-based electroanalytical techniques. We calculated the Michaelis Menten constant from the linear portion of the curve and found that this sensor has a higher affinity to Hydrogen peroxide than other available enzymatic sensors, with a ${K}_{m}^{app}$ of 19.44 µM. The curvature of the microbubble provides more surface area for the embedded heme sites, resulting in signal enhancement without using other transducing elements. These results highlight that the promising H.O.S.T. technology can pave the way for a new generation of H_2_O_2_ biosensors for early, reliable, cost-effective cancer diagnostics. The comparison studies of native hemoglobin microbubble-modified electrodes with HOST (as shown in Figure 6) exhibited a 3.5 times increase in the signal using the Cyclic voltammetry method and a 7.5 times increase in the output signal using the Electrochemical Impedance spectroscopy method. This signal enhancement is attributed to multiple heme sites housed within the hemoglobin microbubble, which are key sensing elements that participate in detecting the expressed hydrogen peroxide. This shows that even without the use of additional transducing elements, our developed sensor has superior performance compared to the current native hemoglobin-based hydrogen peroxide biosensors.

## Results and discussion

### Characterization of microbubbles

**Figure 2:**
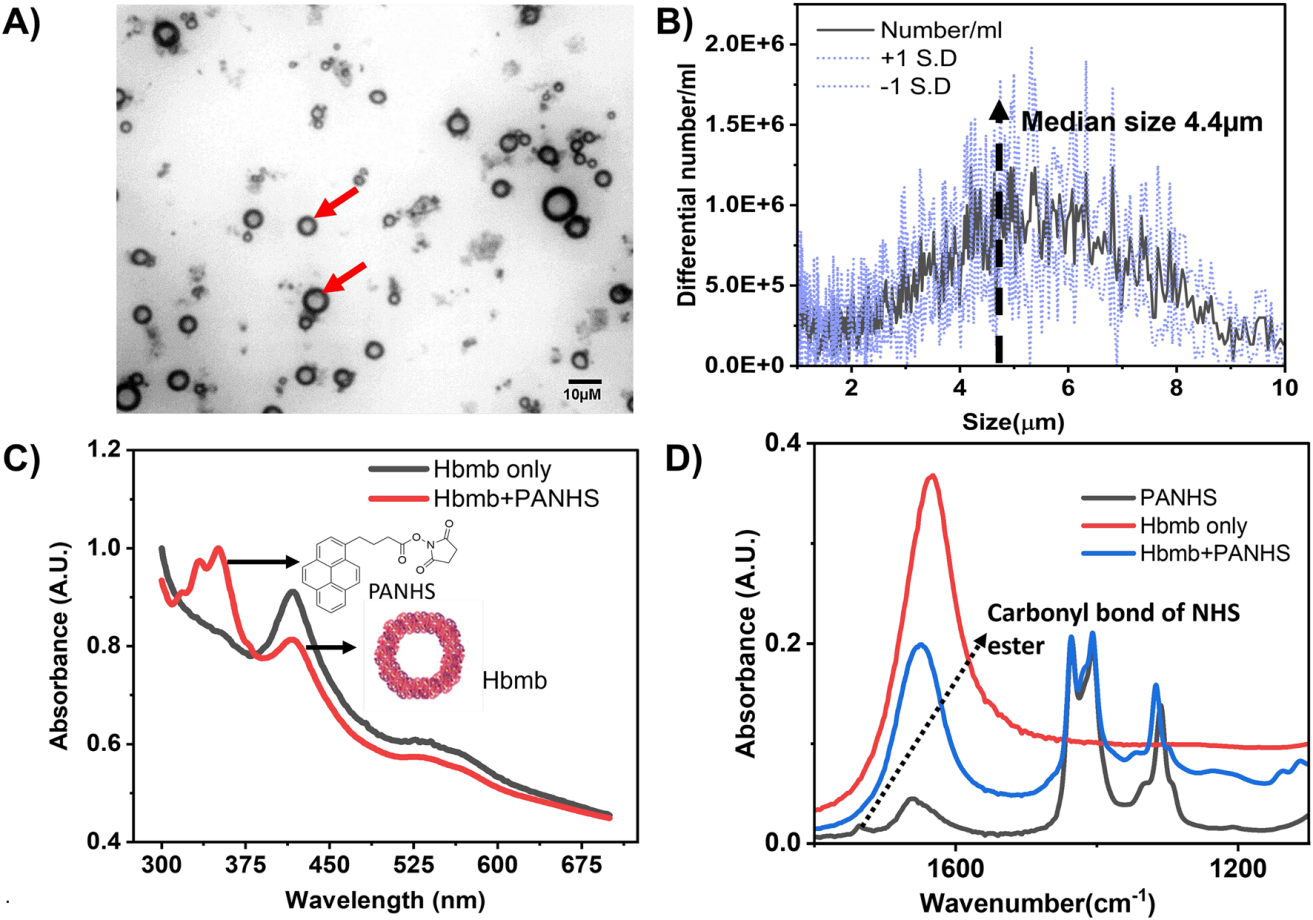
(**A**) Brightfield microscopy of Hemoglobin microbubbles, (**B**) Size distribution of synthesized microbubbles determined using Coulter Counter Method, (**C**) U.V. vis absorbance spectroscopy of Hemoglobin microbubble solution alone (black) and with the crosslinker P.A.N.H.S. (**D**) ATR- FTIR absorbance spectra of the crosslinker PANHS (Blank) Hemoglobin only (Red), Hemoglobin and PANHS (Blue). Partially created using Biorender.com.

Microbubbles are gas-filled micron-sized particles that are stabilized using protein, polymer, or lipid particles. The shell material prolongs the lifetime of the microbubble by reducing the surface tension and gas diffusion^18^ In this study, we use hemoglobin-stabilized air-filled microbubbles that were produced using The sonochemical method of microbubble production, which ensures high yields and has been used to produce protein microbubbles^19–21^. In sonochemical synthesis, high-intensity ultrasound generates superoxides, which oxidize the cysteine bonds in the protein to form disulfide bonds, thus surrounding the gas core. The experimental section has discussed the formulation of the hemoglobin microbubbles. Brightfield microscopy was performed to study the morphology of the prepared microbubbles using a BX50 Upright Microscope (A.C.H. 40X/0.80 ∞/0.17 objective). Figure 2A shows the micrograph of the microbubbles and Fig. 2B indicates the size distribution of hemoglobin microbubbles measured using the Coulter Counter method. The total yield of the microbubbles with this method was 1.9 ± 0.82 e8 Microbubbles/mL of solution. In this way, air-filled hemoglobin microbubbles with a median diameter of 4.4 µm ± 2.1 µm were achieved for developing the sensor stack to demonstrate the detection of H_2_O_2_.

### Validation and optimization of binding chemistry

As described in the experimental section, the hemoglobin microbubbles (Hbmb) were anchored to the sensor's working electrode via a strong P.A.N.H.S. crosslinker. To validate the immobilization of the microbubbles on the working electrode with the P.A.N.H.S. crosslinker, we used Attenuated Total Reflectance Fourier Transform Infrared Spectroscopy (ATR-FTIR) and UV–Vis spectroscopy. Figure 2C shows the UV–Vis spectroscopy spectra for a solution of Hb microbubbles (black line) and a 1:1 cocktail of Hb microbubbles conjugated to P.A.N.H.S. (red line). The Hb microbubble solution was prepared in nitrogen-saturated PBS buffer to minimize the interference from ambient oxygen for successful binding. The pyrene moiety of the P.A.N.H.S. linker exhibits the characteristic three peaks observed in the visible region (310–350 nm, Duhamel, 2005). The UV–Vis spectra show the hemoglobin protein's distinct Soret peak and Q bands at 410 nm and 555 nm, respectively, for the Hbmb and Hbmb-PANHS conjugate. This experiment confirmed that the interaction of hemoglobin with P.A.N.H.S. does not result in the denaturation of the hemoglobin, as the Soret peak and the Q bands are not affected by cross-linking. Figure 2D) shows the ATR-FTIR absorbance spectra of the P.A.N.H.S. crosslinker (black line), the Hb microbubble (red line), and the Hb microbubble bound to the P.A.N.H.S. crosslinker (blue line) in the range of 2400–400 cm^−1^., the peaks between 1200–1500 cm^−1^ are characteristic of the P.A.N.H.S. structure. The pyrene moiety of the P.A.N.H.S. linker is linked to the carbon working electrode using irreversible π – π stacking. The primary NH_2_ groups in the Hb microbubble protein substitute the N.H.S. moiety of the crosslinker through a nucleophilic attack, forming an amide bond with the hemoglobin protein^22^.The suppression of the peak at 1736 cm^−1^ validates the successful conjugation of the P.A.N.H.S. linker molecules with the prepared Hb microbubbles. Optimization studies were performed using ATR-FTIR to select the incubation time of the Hb microbubble for the successful formation of stable bonds with the P.A.N.H.S. crosslinker. The optimal incubation time was 90 min, as discussed in the supplementary Figure S5. Supplementary Table T2 lists the expected and measured peaks for the spectra in detail.

### Electrochemical characterization of the modified electrodes

**Figure 3:**
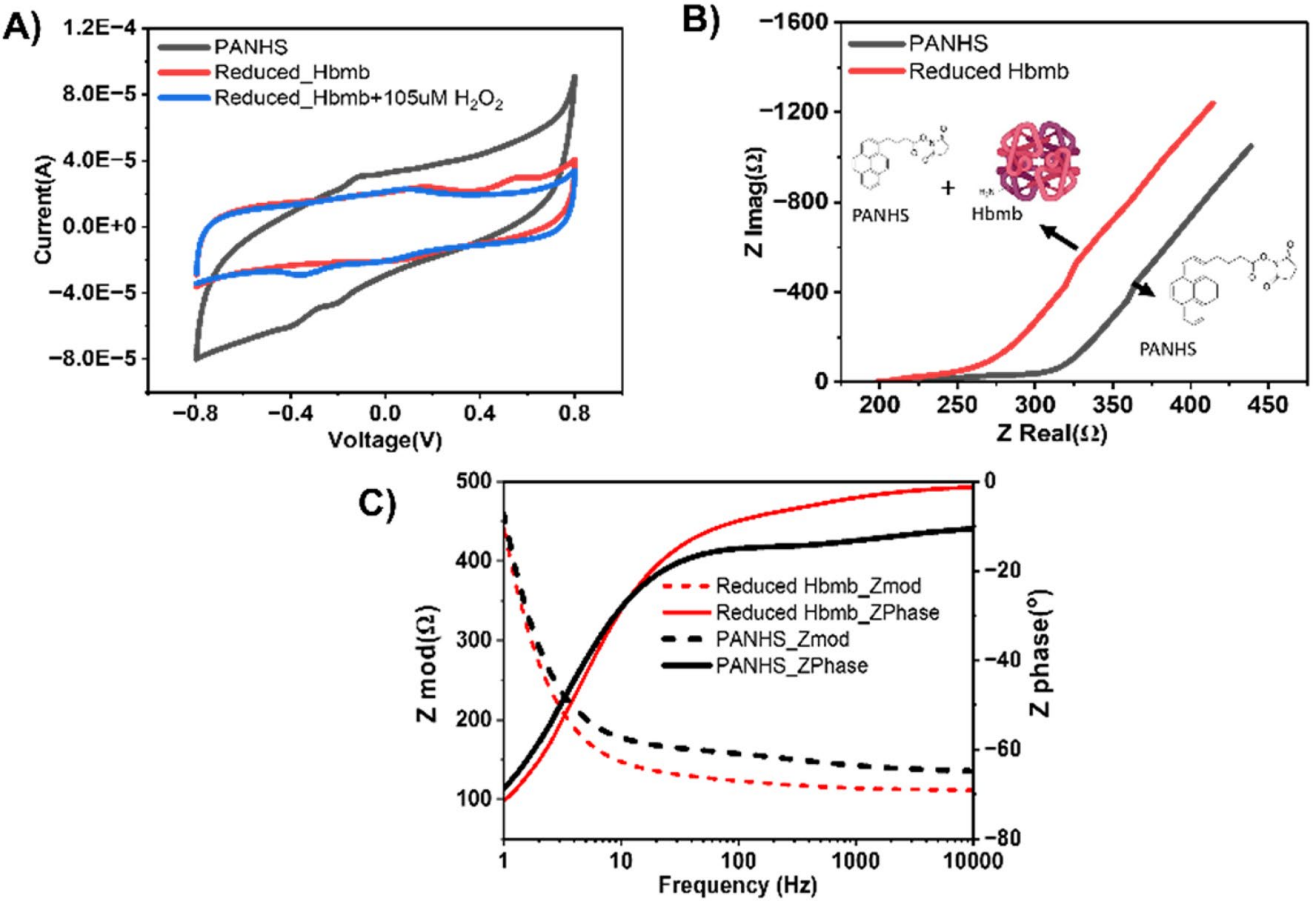
(**A**) Cyclic Voltammetry plot showing electrode modification, CV of Reduced Hbmb/PANHS/SPCE in Nitrogen saturated PBS (red) with 0 µM Hydrogen peroxide, Reduced Hbmb/PANHS/SPCE with the highest dose(105 µM) of H_2_O_2_ (blue). (**B**) Nyquist plot of PANHS/SPCE (black), Reduced Hbmb/PANHS/SPCE (red). Figure (**C**) Bode plot of PANHS/SPCE (black), Reduced Hbmb/PANHS/SPCE (red). Partially created using Biorender.com.

The baseline characterization of the Hbmb-modified electrodes was done using D.C.- and A.C.-based established electroanalytical methods of Cyclic Voltammetry^22–25^, Square wave Voltammetry, and E.I.S. Figure 3A shows the Cyclic voltammetry measurements taken from − 0.8 to 0.8 V at a scan rate of 100 mv/s. The redox reaction between the immobilized Hb microbubble and H_2_O_2_ molecules has been shown in Fig. 1. Initially, the Heme groups in the Hb microbubbles are reduced to the Fe^2+^ state using a reducing agent consisting of sodium dithionite and sodium sulfite. When H_2_O_2_ is present in the sample at the sensor input, it reacts with the Heme site, and here, the ferrous (Fe^2+^) atom undergoes a reduction and oxidizes to the ferric state, i.e., Fe^3+^. When hemoglobin microbubble interacts with the highest dose of hydrogen peroxide, the oxidation peak shifts to 0.13 V, and a single reduction peak occurs at − 0.4 V, which is consistent with the observations of Zhang et al., who observed the reduction peak at around -0.35 V for a similar study using Hemoglobin on gold/graphene chitosan nanocomposite^26^. Thus, the reduction peak was chosen for subsequent Hydrogen Peroxide calibration. The optimal scan rate for CV analysis was selected as 100 mV/s to reliably characterize the electrochemical reaction kinetics and the diffusion of the species from the electrolyte to the electrode. The scan rate was chosen as it provided good reaction equilibrium and optimal detection time for the analyte^27^. The scan rate optimization experiment has been described in detail in Figure S4. Both reduction and oxidation peaks are proportional to the scan rate, indicating characteristic reversible surface-confined electrochemical behavior^28^. E.I.S. experiments were performed to study the subtle changes at the electrode–electrolyte interface due to H_2_O_2_ interaction to the reduced Hbmb. A small A.C. perturbation of 10 mV R.M.S. was applied at the working electrode for a frequency sweep between 1 Hz and 100 kHz, and the resulting impedance was studied at the output to characterize the electrical double layer (EDL) at the electrode–electrolyte interface. The Nyquist plot (imaginary component, i.e., ZImag versus the real component of impedance, i.e., ZReal) in Fig. 3B shows how the E.I.S. response changes due to the conjugation of the Hb microbubble with the self-assembled monolayer of the P.A.N.H.S. crosslinker at the working electrode. The plot is divided into different regions affected by elements of the equivalent Randle’s circuit^29,30^ (described in supplementary Figure S1). Figure 3C shows the Bode magnitude (dotted lines) and Bode phase (solid lines) plots for the P.A.N.H.S. crosslinker (black lines) and P.A.N.H.S. conjugated to the Hbmb (red lines) over the entire frequency range. The conjugation of the Hbmb molecules to the P.A. N.H.S. layer at the interface creates a capacitive modulation of the dielectric constant at the EDL, and a reduction in the imaginary component of impedance (Z_Imag_) was observed. Maximum capacitive behavior was observed at < 15 Hz (as indicated by a negative impedance phase value). Thus, 5 Hz was chosen as the optimal frequency for E.I.S. for subsequent sensor calibration.

### Sensor calibration for hydrogen peroxide detection using cyclic voltammetry, electrochemical impedance spectroscopy, and square wave voltammetry

**Figure 4:**
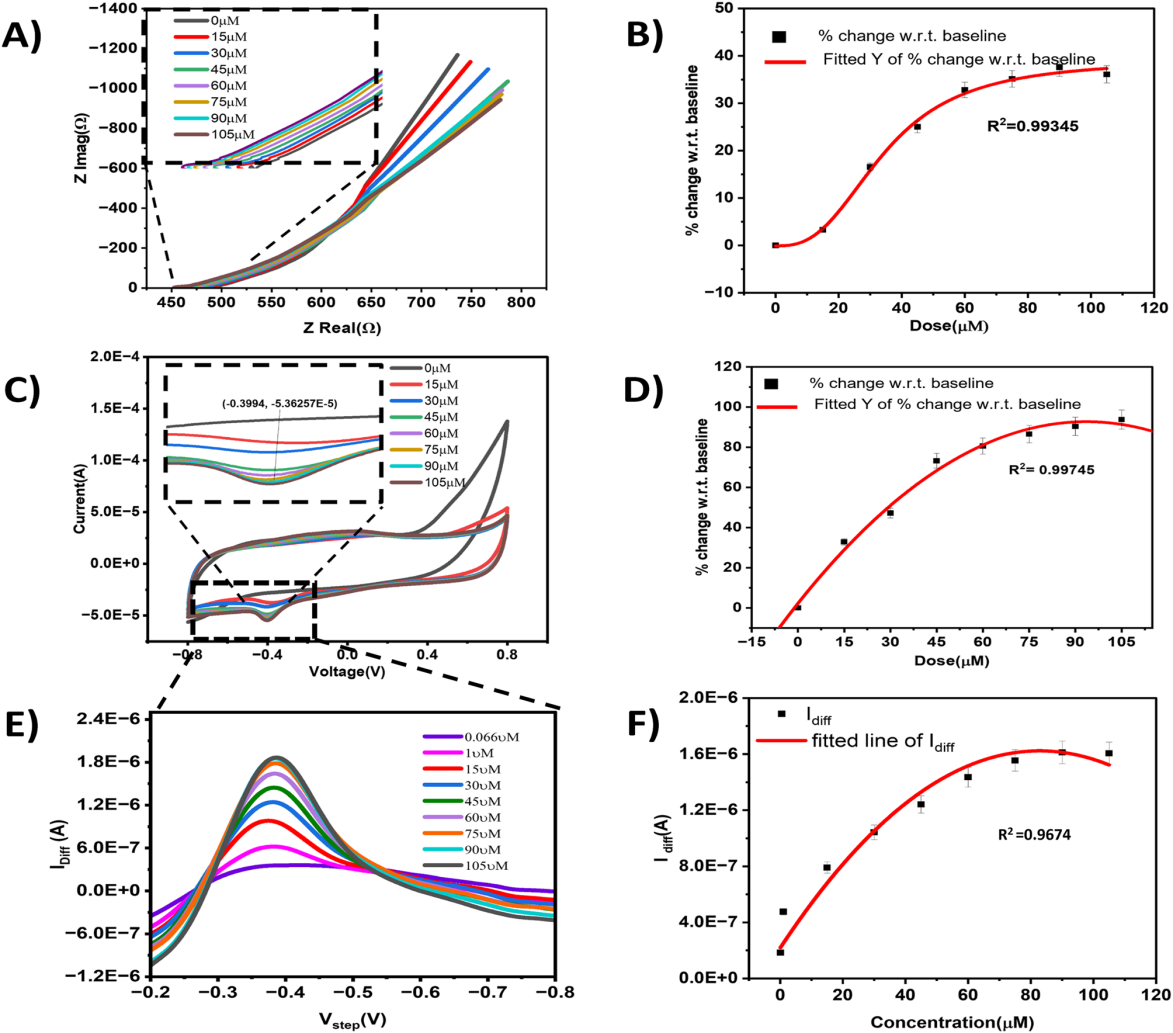
(**A**) Response of sensor, for the concentration of Hydrogen Peroxide between (0 µM to 105 µM), (**B**) corresponding calibration curve showing % in Z_Imag_ at 5 Hz with respect to Baseline with R^2^ = 0.99345 (**C**) Calibration dose response of sensor using CV method Response of sensor, for the concentration of Hydrogen Peroxide between (0 µM to 105 µM). (**D**) corresponding calibration curve showing % change in current taken at 0.39 V with respect to baseline (0 µM), with R^2^ = 0.9975. (0 µM). (**E**) calibration dose response using Square wave voltammetry between (66 nm–100 µM) (**F**) Corresponding calibration curve showing plot of I_diff_ versus V_step_ and the R^2^ = 0.9645.

The Fig. 4A shows the E.I.S. dose–response curve of the developed sensor as a Nyquist plot. As seen in the figure, a dose-dependent response is obtained for varying levels of H_2_O_2_ ranging from 15 to 105 μM. The inset zooms in to show the dose-dependent response for the mid to high-frequency ranges where capacitive behavior is observed. Figure 4B indicates the sensor's calibration dose–response curve for E.I.S. analysis. The percent change in the imaginary component of impedance (Z_Imag_) relative to the blank/baseline dose (i.e., 0 μM of H_2_O_2,_ also known as the “Zero dose” or “Z.D.”) at a frequency of 5 Hz was used for calibrating the sensor. 4-Parameter Logistic curve fitting was done to study the relation between the input analyte concentration and the relative modulus of impedance (% change in Z_Imag_ relative to Z.D.). The sensor shows a high correlation coefficient of R^2^ = 0.99745 for E.I.S. This calibration establishes the dynamic range of 15–105 μM for interpreting hydrogen peroxide dose concentrations and demonstrates the sensor's high sensitivity. Cyclic voltammetry (CV) was performed for additional validation of the stability and sensitivity of the sensor for the H_2_O_2_ dose concentrations from 15 to 105 μM in 1X PBS. The results are shown in Figs. 4C,D. The cyclic voltammogram Fig. 4C indicates a significant enhancement of the reduction current with increasing H_2_O_2_ concentrations. This signal enhancement is due to the multiple heme site on the bubble surface and the more exposed electroactive center.” The reduction current peak occurs at − 0.4 V, which relates to the reduction of the added H_2_O_2_ and oxidation of the Heme site from the ferrous to the ferric state, as described previously. The inset shows that the increasing concentration of H_2_O_2_ increases the reduction current, which mediates charge transfer at the sensing electrode. The calibration curve of this peak reduction current at − 0.4 V with H_2_O_2_ dose concentrations is plotted in Fig. 4D. Polynomial curve fitting was done to study the relation between the input analyte concentration and the relative change in current (% change in Current vs. Z.D.). The sensor shows a high correlation coefficient of R^2^ = 0.99345 for CV. This way, highly sensitive hydrogen peroxide detection and quantification was achieved using CV and E.I.S. To Corroborate the reduction Peak at − 0.4 V found using Cyclic voltammetry, we also performed Square .

wave voltammetry. Figure 4E shows dose-dependent increase in I_diff_ at − 0.4 V for the doses between 66 nM and 100 µM. In Fig. 4F Polynomial curve fitting was done to study the relation between the input analyte and the Differential Current measure at − 0.4 V. The R^2^ obtained for the fitting was 0.9645. We also calculated the apparent Michaelis -Menten constant ${K}_{m}^{app}$ which is an important parameter as it reflects the enzyme–substrate reaction kinetics. We calculated the ${K}_{m}^{app}$ using the Linweaver Burk plot (Figure S1) and used $\frac{{1}}{{{\text{I}}_{{{\text{SS}}}} }}{ = }\frac{{{\text{K}}_{{\text{m}}}^{{{\text{app}}}} }}{{{\text{I}}_{{{\text{max}}}} {\text{*C}}}}{ + }\frac{{1}}{{{\text{I}}_{{{\text{max}}}} }}$ Where I_ss_ is the Steady State, ${\text{K}}_{{\text{m}}}^{{{\text{app}}}}$ is Michelis Menten Constant, C is the substrate Concentration and I_max_ is the maximum current. The slope and intercept were used to calculate the ${K}_{m}^{app}$ 19.44 µM, which is much lower than the previously reported values of 235 µM and 69 µM^31–33^. Higher affinity towards hydrogen peroxide was attributed to the crosslinked hemoglobin in microbubble form, hence more heme sites interacting with the Hydrogen peroxide molecules.

### Analysis of non-specific binding and effect of interferents using CV and E.I.S.

**Figure 5:**
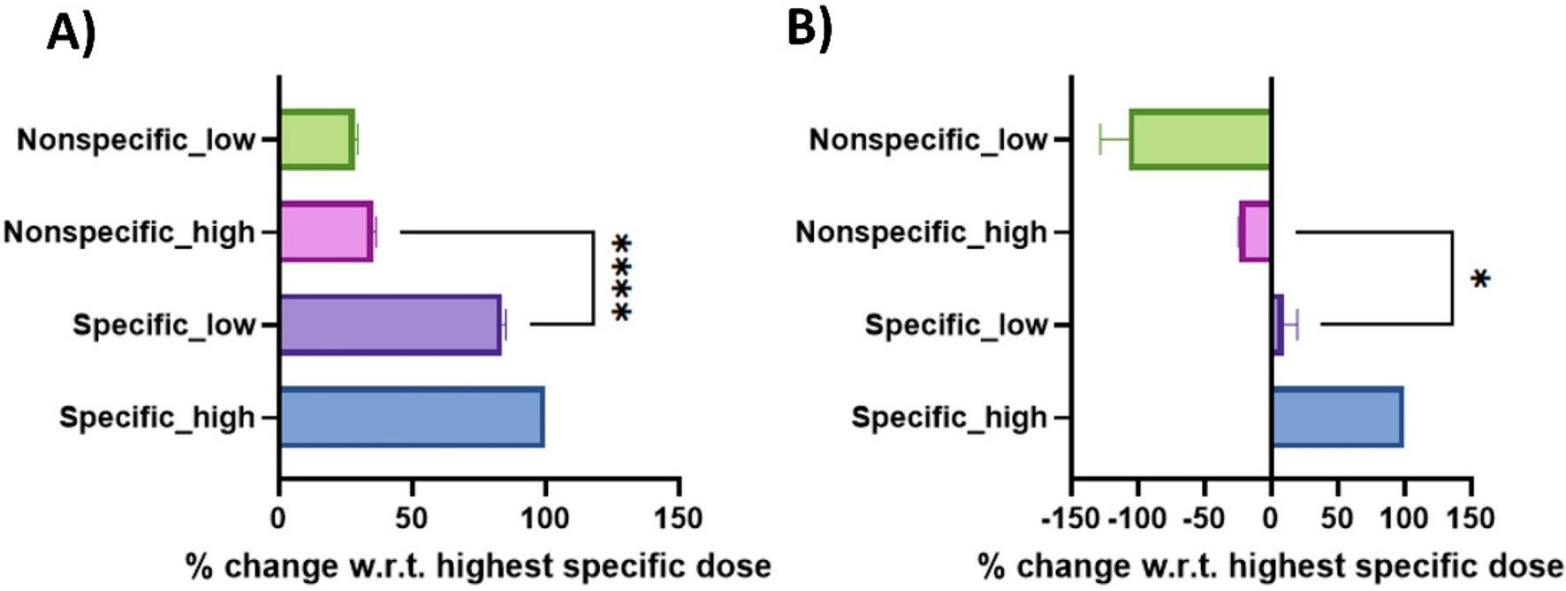
(**A**) Analysis of the effect of interferents using voltammetric sensing of hemoglobin microbubble modified electrode, specific doses consisting of Hydrogen peroxide (15 µM, Low dose), (45 µM, High dose), Non-Specific Doses contain a cocktail of common interferents in hydrogen peroxide sensing such as Glucose and Ascorbic Acid in concentration as follows (15 µM A.A., 15 µM Glu, Low dose), (45 µM A.A., 45 µM Glu, High Dose). (**B**) Effect of interferents using Impedimetric sensing of hemoglobin microbubble-modified electrode.

To evaluate the specificity of the novel microbubble-based capture probe towards H_2_O_2_ in the presence of common cross-reacting biomolecules, an interference study was conducted with the lowest and the highest concentrations of non-specific molecules like Glucose and Ascorbic acid using both CV and E.I.S. methods as described in the previous section. A 1:1 cocktail solution of Glucose (Glu) and Ascorbic acid (A.A.) was prepared for this study (Low dose: 15 µM A.A., 15 µM Glu; High Dose: 45 µM A.A., 45 µM Glu). The interference measured from varying non-specific molecules showed a statistically significant difference compared to specific H_2_O_2_ dose response (Low dose: 15 µM; High dose: 45 µM). Figure 5A displays specific and non-specific CV responses as a percentage change in current. Figure 5B shows the specific and non-specific E.I.S. response as a percentage change in the imaginary component of impedance (Z_Imag_). The developed sensor was able to distinguish specific H_2_O_2_ concentration from cross-reacting molecules significantly (*p* < 0.0001) for CV and (*p* = 0.043) for E.I.S. when spiked with the highest physiologically relevant concentration of each.

### Sensor performance comparison versus native hemoglobin

**Figure 6:**
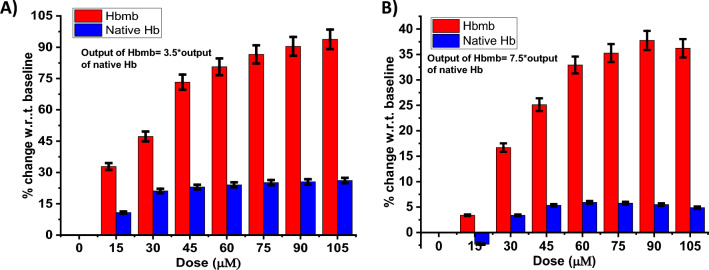
(**A**) Sensor performance comparison for hydrogen peroxide detection of HOST (Red Bar) with Sensor performance of Native hemoglobin modified electrode (Blue Bar) cyclic voltammetry method (**B**) Sensor performance comparison of HOST with Native hemoglobin modified electrode using EIS method.

H.O.S.T. uses hemoglobin microbubbles as the affinity capture probe for the preferential binding of the H_2_O_2_ analyte molecules instead of using plain native hemoglobin^34^. It was hypothesized that a single hemoglobin microbubble could house more heme sites (multiple hemoglobin tetramers are crosslinked via disulfide bridges to form the shell of the microbubbles^35^, versus a single native hemoglobin molecule which contains four heme sites^36^, using a microbubble capture probe would allow for enhanced sensor metrics. Owing to the increased surface area and the presence of more heme sites, the probability of interaction of an H_2_O_2_ with a heme site increases, resulting in greater electroactive surface concentration and signal amplification without the need to add a redox tag or additional transduction elements in the assay such as Nafion^37^. To test this hypothesis, we compared the sensor output with plain native hemoglobin modification at the working electrode versus that for hemoglobin microbubble modified working electrode (H.O.S.T.) using both E.I.S. and CV methods. Figure 6A H.O.S.T shows a wide output signal range of 32–93% for CV, whereas the native hemoglobin-modified electrode showed an output signal range of (10.7 to 26%) and Fig. 6B 3.3–36% for E.I.S., In the Native hemoglobin-modified electrode, the output signal range was (− 2 to 4.8%). Thus, H.O.S.T. shows a lower limit of detection and wider output dynamic range for hydrogen peroxide detection. In this way, the improvement in sensor performance due to the novel microbubbles was validated.

### Sensor performance testing in cell lysates

**Figure 7:**
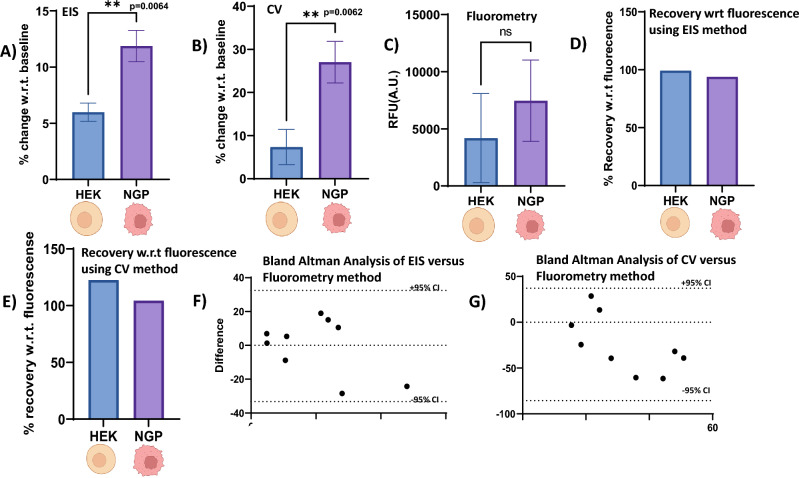
Sensor performance evaluation for Hydrogen Peroxide detection in Cell Lysates-NGP cells (Neuroblastoma) and H.E.K. (non-Cancerous). (**A**) using E.I.S. method, (**B**) using CV method, (**C**) fluorometric method using a commercially available (**D**, **E**) Percentage recovery Hydrogen peroxide in lysate, with respect to Fluorescence, using CV(7D) and E.I.S. methods (**E**) hydrogen peroxide Assay kit. (**F**) Bland Altman Analysis of H.O.S.T (EIS method) and EpiQuik Hydrogen peroxide assay kit. (**G**) Bland Altman Analysis of H.O.S.T (CV method) EpiQuik Hydrogen peroxide assay kit. Partially created using Biorender.com.

Cell lysates were collected from N.G.P. (Neuroblastoma or Cancerous) Leibniz Institute DSMZ-GmbH, Braunschweig, Germany, and H.E.K 293(American Type Culture Collection, Manassas, VA), (non-Cancerous) cells, preparation of cell lysate is described in the supplementary document. Figures 7A,B show the respective E.I.S. and CV sensor response comparison for N.G.P. versus H.E.K. cell lysates. As expected, in both methods, the sensor response for N.G.P. was significantly higher than that for H.E.K. This is because N.G.P. cells, being cancerous, release a higher concentration of R.O.S., resulting in high H_2_O_2_ levels. Using Student’s *t* test (unpaired, two-sided, α = 0.05), a *p* value of 0.0064 for E.I.S. and 0.0062 for CV was obtained. In this way, the diagnostic efficacy of the developed sensor for R.O.S. detection was validated in real cell lysate samples.

### Sensor performance comparison with commercial fluorescence assay in real cell lysates

To compare and benchmark the performance of H.O.S.T., we used EpiQuik™ Hydrogen Peroxide Assay Kit, a commercially available fluorometric hydrogen peroxide Assay kit (n = 3). The protocol for preparing the cell lysates and the steps for measuring hydrogen peroxides using EpiQuik™ have been discussed in detail in the supplementary information. Figure 7C shows the signal response obtained from the commercial kit for H.E.K. and N.G.P. cell lysate samples. Our developed sensor outperforms the commercial kit, which shows no statistical significance between H_2_O_2_ levels for the cancerous and non-cancerous cell lysate samples (*p* = 0.159), whereas our sensor shows a statistically significant difference for the two groups (*p* = 0.0064 for E.I.S. and *p* = 0.0062 for CV). From the EpiQuik Hydrogen Peroxide Assay kit, the H_2_O_2_ levels for the H.E.K. and N.G.P. cells were calculated using regression analysis, which was found to be 21 µM and 9 μM, respectively. Using the regression analysis of the 4-PL calibration curves obtained for E.I.S. and polynomial curves for CV discussed in Sect. 3.4, the H_2_O_2_ levels were recovered for each method. Using the commercial kit as a benchmark, the H_2_O_2_ levels of the lysate samples were recovered (discussed in detail in the supplementary information), as shown in Fig. 7D for E.I.S. and Fig. 7E for CV methods. For E.I.S., a 104% average recovery from N.G.P. cell lysate and 122% from H.E.K. cell lysate was achieved.

In contrast, the average recovery for CV is 93% from N.G.P. cell lysate and 99% from H.E.K. cell lysates, which lies within the acceptable recovery range (80–120% of the expected concentration) as per the Clinical Laboratory Standards Institute(C.L.S.I.)^38^. Bland–Altman analysis was performed to compare the response of H.O.S.T. (both E.I.S. and CV) with that of the commercial fluorescence kit. Bland–Altman analysis for the values obtained from our sensor and EpiQuik TM Hydrogen peroxide Assay kit from spiked H_2_O_2_ levels in cell lysate is displayed in Fig. 7F for E.I.S. and 7G for CV analysis. The bias value is − 0.37 for E.I.S. and − 24.26 for CV, indicating systematically similar results produced by the two tests. All the measurements fall within the 95% confidence intervals (± 1.96 S.D.), indicating agreement in the results of the two measurements. Overall, this analysis suggests the agreement between H.O.S.T. and EpiQuik™ H_2_O_2_ Assay values are within the clinically relevant ranges despite the limited number of samples tested (n = 3).

Here, we have developed a novel hemoglobin microbubble Oxidative stress sensor, which was characterized using electrochemical impedance spectroscopy, Cyclic Voltammetry, and Square Wave Voltammetry and could reliably detect the presence of hydrogen peroxide in the cell lysate sample and distinguish between Cancer and normal cells based on presence of higher levels of hydrogen peroxide in the cancer cells, we corroborated the data we got using our technique by comparing it with a commercially available hydrogen peroxide detection kit, that uses fluorescence detection method to quantify the presence of hydrogen peroxide, in the cell lysates.

## Methods

### Materials and reagents

Lyophilized Human Hemoglobin, N acetyl DL Tryptophan, Sodium Sulfite, Hydrogen peroxide, the solution was prepared fresh before each experiment, Pyrene butyric acid N Hydroxy Succinimide Ester (P.A.N.H.S.), Dimethyl Sulfoxide (DMSO), Ascorbic acid and Glucose were purchased from was purchased from Sigma Aldrich (St. Louis, MO).

### Preparation of hemoglobin microbubbles using the sonochemical method

Hemoglobin solution (10 mg/ml) was prepared by dissolving 400 mg lyophilized human Hemoglobin (Sigma Aldrich, St. Louis, MO) in 40 of 1X PBS containing 10% glycerol(v/v) and 10%propylene glycol, (v/v),100 mg of N acetyl DL Tryptophan was dissolved in 10 mL of PBS containing 10% glycerol and 10%propylene glycol, then 10 mL was added to the hemoglobin solution. The introduction of tryptophan helped to improve the yield of protein microbubbles by increasing the unfolding of the protein structure for better shell stability^39^. The hemoglobin-tryptophan solution was preheated to 50 °C and moved into a sound-proof enclosure housing a Branson 450 Sonifier Analog cell disrupter (Danbury, CT). The ½ inch ultrasonic horn was immersed at the air–liquid interface. The solution was sonicated at 100% amplitude for 5 s to form air-filled hemoglobin bubbles, then immediately cooled in an ice bath. The microbubbles were transferred to four 10 mL Luer tip syringes, B.D. (Franklin Lakes, NJ), and centrifuged at 300 relative centrifugal force (R.C.F.) for 3 min in a Bucket Centrifuge Model 5804R, Eppendorf (Hauppauge, NY) to collect and concentrate buoyant microbubbles, separating them from residual unincorporated hemoglobin remaining in the infranatant. The microbubbles were consolidated into a 10 ml syringe and resuspended with) 0.1 M PBS. The step mentioned above was repeated until the infranatant was clear. Washed bubbles were concentrated for further experiments. Figure 1 shows the pictorial representation of the Hemoglobin microbubble synthesis. In-depth synthesis is included in the supplemental Figure S7.

### Physical characterization of microbubbles

The protocol for synthesizing the novel hemoglobin microbubbles has been discussed in detail in the supplementary information. After preparing the microbubble solution, the Coulter Counter method was used to measure particle size distribution and concentrations of the microbubbles. A 2 μL sample of microbubbles was diluted in 10 mL of isotone II within a 25 mL cuvette and characterized using a Multisizer 4e Coulter Counter (MS4), Beckman Coulter (Brea, CA). The samples were measured three times, and the size distributions were averaged. A BX50 Upright Microscope (A.C.H. 40X/0.80 ∞/0.17 objective) was used to visualize the microbubbles for brightfield microscopy. A small square-shaped well was created using high vacuum silicone grease, Dow Corning (Midland, MI). 10 µL of the sample after dilution was placed in the well on a 25 × 75 × 1 mm microscope slide covered with a glass slide cover (Fisher Scientific, Waltham, MA).

### Preparation of hemoglobin microbubble-modified electrode

Screen Printed Carbon electrode (Dropsens, Metrohm, U.S.A.) was used for preparing the electrochemical sensor, with Carbon as the Working electrode, Counter electrode, and silver as the reference electrode. Before fabrication, screen-printed electrode is washed with Isopropyl alcohol Deionized water and nitrogen dried. Pyrene Butyric acid N.H.S. ester (P.A.N.H.S.) crosslinker is prepared by measuring 1 mg of P.A.N.H.S. and adding 250 µl dimethyl sulfoxide, 5 µl of this solution is drop casted on the Working electrode, this crosslinker-modified surface is incubated for 90 min inside the faradaic cage under constant nitrogen supply, the surface is washed three times to remove any unbound P.A.N.H.S. linker, then 5ul of prepared microbubble solution is added on the Working electrode and again incubated for another 90 min. Hemoglobin microbubbles, prepared sonochemically, is in a ferric state The hemoglobin microbubble solution was reduced to a ferrous state using a reducing agent solution of 13.5 mM Sodium Dithionite (Pfaltz & Bauer, Waterbury, CT)Sodium sulfite (Sigma Aldrich, St. Louis, MO). This way of preparing the modified electrode ensured enhanced signal from the hemoglobin microbubble-modified electrode, through better charge transfer between the heme site and the carbon electrode when the interaction occurs with Hydrogen peroxide and the heme site of the microbubble.

### Characterization of binding chemistry between hemoglobin and P.A.N.H.S. crosslinker

We used ATR FTIR and UV Vis Methods to characterize the binding chemistry to study the interaction of hemoglobin microbubble with P.A.N.H.S. Crosslinker. The samples to be tested consisted of a 1:1 solution of reduced Hemoglobin microbubbles with P.A.N.H.S. solution and 1:1 of reduced Hemoglobin microbubble solution with nitrogen-saturated PBS(Control). The spectra were obtained using Nicolet 6700 FTIR (Thermo Fisher Scientific, MA, U.S.A.).

A total of 256 scans were collected for each spectrum, for a wavelength range of 2400 to 400 cm^−1^, at a resolution of 4 cm^−1^. A spectral absorbance scan was done using UV–Vis spectroscopy (Agilent Synergy Biotek H4 Microplate reader, Santa Clara, CA) between the wavelength 300 nm–700 nm, with increments of 1 nm, under ambient conditions.

### Design of dose–response curves cyclic voltammetry (CV), electrochemical impedance spectroscopy (E.I.S.) and square wave voltammetry (S.Q.W.V.)

Doses of 15–105 µM of hydrogen peroxide were prepared in nitrogen-saturated PBS and stored in the dark. After preparing the working electrode as mentioned above, 60 µL of zero dose (nitrogen-saturated PBS without Hydrogen peroxide) was added and incubated for 1 min before taking the E.I.S. and CV measurements. After the baseline measurements containing 0 µM Hydrogen peroxide solution, 60 µL of doses within the range of 15–105 µM were drop casted as mentioned above. E.I.S. measurements were taken using a potentiostat (A.C. excitation signal of 10 mV R.M.S. and a D.C. bias of − 0.4 V). CV measurements were taken between − 0.8 V and 0.8 V at a scan rate of 100 mV/s with a step size of 2 mV. For Square wave Voltammetry, Measurements were taken between − 0.8 V and − 0.2 V, with a step size of 2 mV, frequency, and Amplitude of 10 Hz and 10 mV, respectively. To further test the limit of detection, Doses of 66 nM to 105 µM were drop casted for plotting the calibration curve using this method. This method was also used to validate the peak obtained with Cyclic Voltammetry at − 0.4 V with the addition of Hydrogen Peroxide. All the electrochemical measurements were taken using Gamry Reference 600 (Gamry Instruments. Warminster, PA).

## Conclusion

In summary, we have developed a novel, label-free/mediator-free biosensor- H.O.S.T. The biosensor uses a novel air-filled hemoglobin microbubble as a capture probe for preferential binding of the target hydrogen peroxide molecules. The developed Hemoglobin microbubble-based biosensor is suitable for real-time monitoring of extracellular H2O2 for clinical research. This technology uses complementary electrochemical detection techniques, Electrochemical Impedance Spectroscopy, and Cyclic Voltammetry for specific and sensitive detection of Hydrogen peroxide in cell lysate samples. The sonochemically prepared hemoglobin microbubble retained the bioactivity through the formulation and immobilization process and showed excellent catalytic activity. We hypothesized that the crosslinked Hemoglobin protein in the microbubble architecture showed better affinity towards Hydrogen peroxide and enhanced signal compared to Native hemoglobin, which can be attributed to more heme sites housed on the microbubble surface, the electroactive center of sonochemically produced hemoglobin microbubbles is also exposed as opposed to that in the native hemoglobin where the electroactive center is buried deep in the enzyme, thus facilitating direct electron transfer by shortening the distance between hemoglobin enzyme and the electrode. Sensitivity and selectivity in target analyte detection were demonstrated using E.I.S., CV, and S.Q.W.V. for the limit of detection at 1 μM Hydrogen Peroxide. High sensitivity and a wide dynamic range were achieved due to enhanced surface area properties compared to a simple native hemoglobin-modified electrode. Selectivity was established through cross-reactivity studies using Glucose and Ascorbic acid as interferents. The diagnostic efficacy of the developed sensor was tested in real cell lysate samples obtained from cancerous and non-cancerous cells. This study also analyzed the sensor performance using Bland–Altman analysis to evaluate the accuracy of sensor calibration and detection of hydrogen peroxide using a commercially available fluorescent hydrogen peroxide detection kit. To our knowledge, this work is the first technological proof of deploying a novel Hbmb for signal enhancement of a .O.C.P.O.C. electrochemical biosensor. Future studies would include testing the efficacy of the developed H.O.S.T. platform for hydrogen peroxide quantification in real human subject samples.

### Supplementary Information

Supplementary Information 1.

Supplementary Information 2.

## Data Availability

Data generated and analyzed during this study is available from the corresponding author upon reasonable request.
