# Supplementary material for: H.O.S.T.: Hemoglobin microbubble-based Oxidative stress Sensing Technology
Source: Sci Rep. 2023 Sep 11;13:14942. doi: 10.1038/s41598-023-42050-z (PMC10495409; doi:10.1038/s41598-023-42050-z)
Supplement: Supplementary file 1 — Supplementary Information 1. [file 41598_2023_42050_MOESM1_ESM.pdf]

H.O.S.T. - Hemoglobin microbubble based Oxidative stress Sensing Technology

Antra Ganguly [a, §], Sugandha Chaudhary [a, §], Shashank R. Sirsi [a], Shalini Prasad [a, *]

[a] Department of Bioengineering, The University of Texas at Dallas, Richardson, Texas 75080, United States

[*] Corresponding author, Shalini.Prasad@utdallas.edu

[§] These authors contributed equally.

Supplementary Information

Supplementary Table 1: Methods of detection of Hydrogen peroxide

**Substrate/ Enzyme or Ligand**	**Dynamic Range**	**LOD**	**Interference Tested**	**Test with whole cell/lysates**	**Contains redox mediators**	**Reference**
**F-MoS2-FePt NCs**	**8-300 µM**	**2.24 µM**	**no**	**yes**	**no**	(Hu et al., 2019)
**SPCE/ Hb-AuNPs**	**3-240 µM**	**4.4 µM**	**yes**	**no**	**no**	(Elewi et al., 2020)
**Gold/ Ferrocenyl methanol(potentiometric)**	**10-1000 µM**	**10 µM**	**no**	**no**	**yes**	(Iwata et al., 2018)
**Gold/11 ferrocenyl Undecanethiol**	**10-1000 µM**	**100 µM**	**no**	**no**	**yes**	(Iwata et al., 2018)
**SPCE/PANHS/Hbmb**	**1-105 µM**	**1 µM**	**yes**	**yes**	**no**	**This work**

Supplementary Table 2: ATR FTIR peak positions and their descriptions.

**Peak Position (cm^{-1})**	**Peak Description**
1452	CH2 Bending
1370	CH3 Bending
1736	Carbonyl stretch of NHS ester in PANHS cross-linker
1659	Amide-II bond

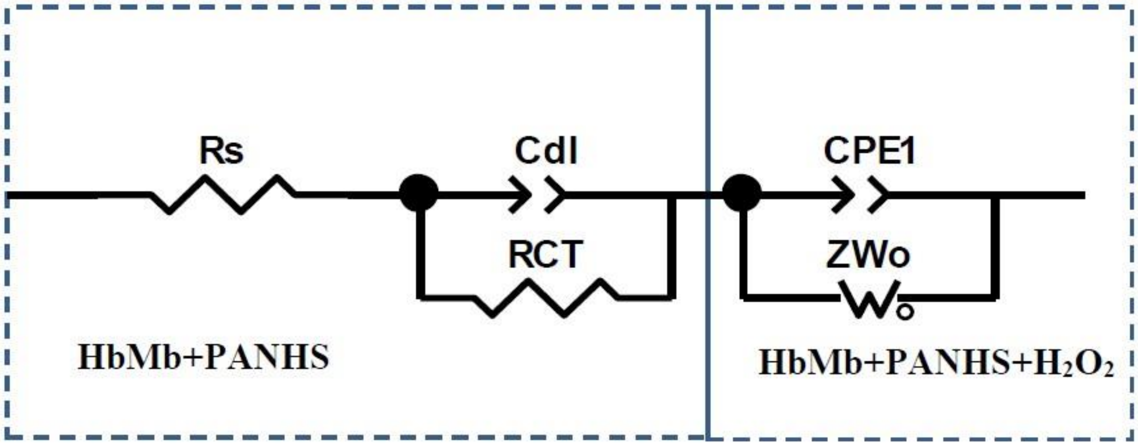

Figure S1: Equivalent Circuit representing the binding interaction between Hemoglobin microbubble modified electrode with Hydrogen Peroxide analyte.

Cyclic Voltammetry characterization of reduced vs. unreduced hemoglobin.

Prior to use, screen printed carbon electrodes were washed with Isopropyl Alcohol and De-ionized water, and Nitrogen dried. 10 mM PANHS crosslinker solution was prepared in DMSO and stored in the dark. 5µL of this solution is drop casted on the working electrode, and the modified electrode is incubated under a constant supply of Nitrogen for 90 minutes. Then 5µL of prepared hemoglobin microbubble solution is drop casted on the working electrode and incubated inside a faradaic cage for another 90 minutes under a constant supply of nitrogen gas. The reduced hemoglobin microbubble-modified electrode was prepared the same way as mentioned above, adding 2µL of freshly prepared reducing agent consisting of sodium dithionite and sodium sulfite before the electrochemical experiment. Cyclic voltammetry measurements were taken from -0.8V to 0.8V and a scan rate of 100mV/s. We further tested the Reduced Hbmb modified electrode characteristics in Oxygenated and PBS that was nitrogenated for 30 minutes to remove oxygen, we found that there was no peak observed at 0.4V with Deoxygenated PBS as shown In fig S2B) which is used as the reduction potential peak for Hydrogen peroxide detection. We used deoxygenated PBS for all experiments including calibration, interference and cell lysate samples.

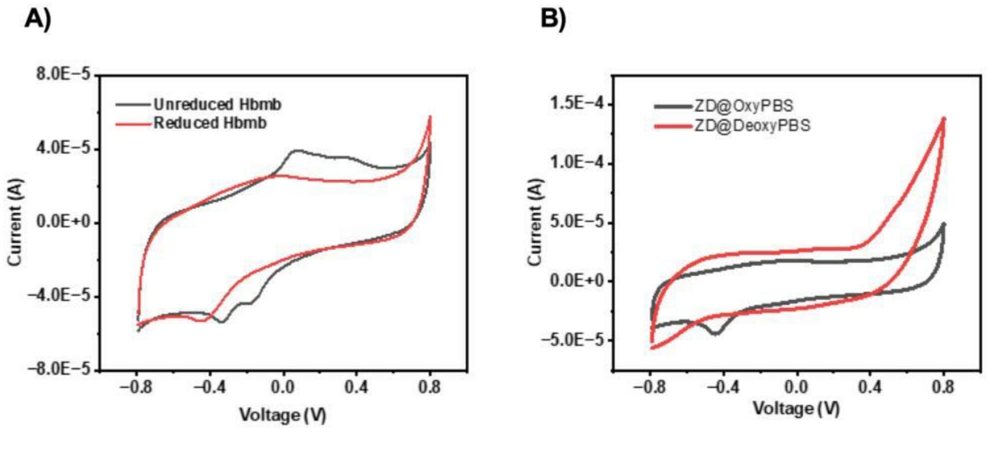

Figure S2: A) Cyclic voltammetry characterization of reduced versus unreduced hemoglobin. **B)** H.O.S.T sensor in oxygenated and nitrogen saturated (deoxygenated) PBS.

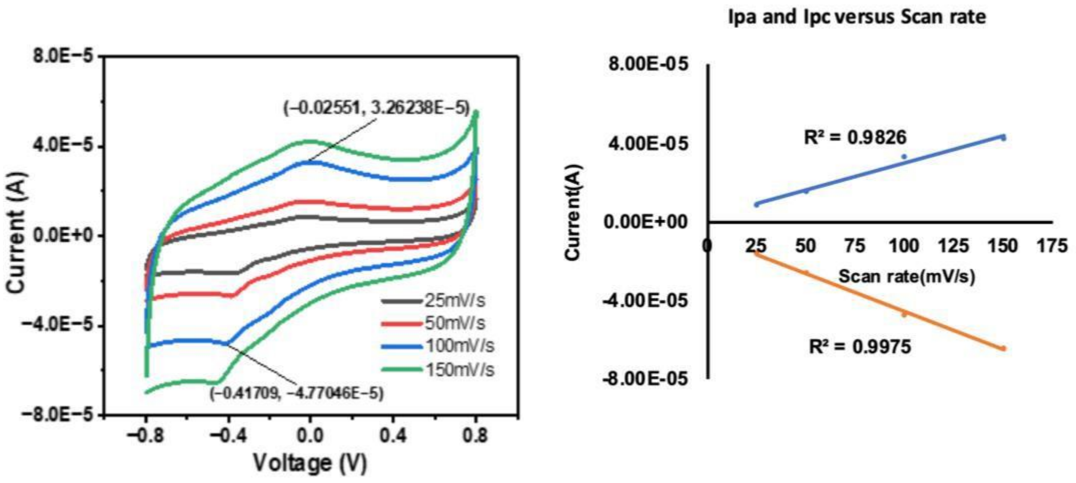

Figure S3: Scan rate study of H.O.S.T sensor between (25mV/s-100mV/s), taken with highest dose of Hydrogen peroxide. The inset shows that the anodic peak current at 0.025V linearly increasing with the increase in scan rate, and cathodic current taken at (0.40V) also increases linearly.

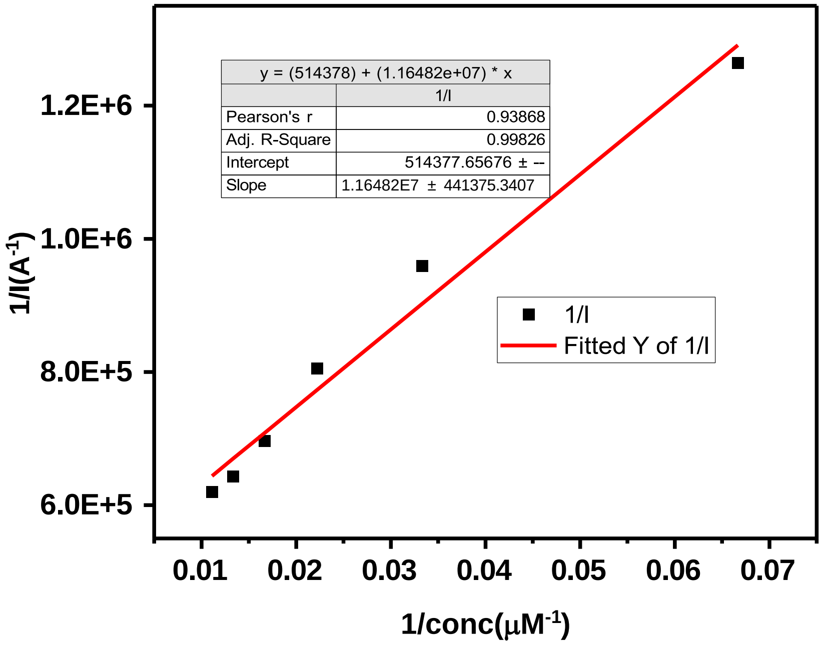

Figure S4: Linweaver Burk plot used to show the linear region of the reciprocal of current versus the reciprocal of the concentration. The slope and intercept were used to calculate the Km which was found to be 19.44 micromolar.

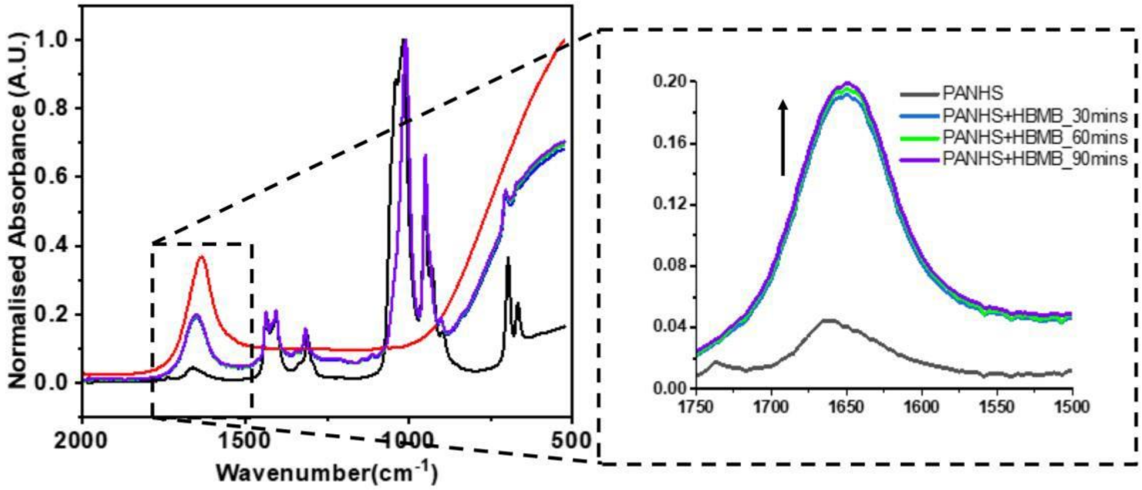

Figure S5: FTIR time Study for optimization of crosslinker (PANHS) binding with hemoglobin microbubble, for effective sensor modification with hemoglobin microbubble. As the time increases, the absorbance amplitude, of amide (II) at 1674nm increases, absorbance amplitude of the amide bond formed after crosslinking increased incrementally after every 30 minutes of incubation, indicating that the bond formation occurs between hemoglobin microbubbles and NHS ester of crosslinker as time increases.

Percentage recovery for determining the amount of hydrogen peroxide recovered from the cell lysates using the Electrochemical methods (CV and EIS) and benchmark method (Fluorescence detection using EpiQuik Hydrogen peroxide Assay Kit). The recovery percentage was calculated using the Equation below.

$$\frac{\boldsymbol{Value\ measured(using\ CV\ or\ EIS\ method)}}{\boldsymbol{Values\ obtained\ using\ benchmark\ method}} * \mathbf{100}$$

Preparation of cell culture and lysates

N.G.P. and H.E.K 293 Cells were harvested at 90% confluency. A total of 1×10^6 cells were obtained for healthy and cancer cell lines. 20µL of this solution is taken to be counted using a hemocytometer. The cell suspensions of both cell lines were then centrifuged at 1500 rpm for 5 minutes to form a pellet. The media solution was discarded, the pellet was resuspended with 0.5mL of ice-cold PBS, and then 500µg of 1.4mm ceramic beads (MO BIO Laboratories, Carlsbad, CA) were added. The solution was mechanically agitated using a Vial mix dental amalgamator (Bristol-Myers Squibb Medical Imaging (New York, NY) for 45 seconds, in these steps, the cells were lysed, and the cell contents including the analyte of interest, were obtained, the homogenate was centrifuged at 10,000 rpm for 5 minutes at 4 ° Celsius to remove the debris. The supernatant contained the analyte of interest.

Hydrogen peroxide measurement using commercial Fluorescence Assay (EpiQuik kit)

To verify the presence of hydrogen peroxide in the lysates, EpiQuik In-Situ and Ex-Situ Hydrogen Peroxide assay kit was obtained (EpigenTek, Farmingdale, NY), and a standard curve was made using the vendor’s protocol. To determine the level of hydrogen peroxide in cell lysate, we first

prepare the Fluorescence Development Solution by adding 1µL of Hydrogen peroxide assay probe and 1µL of Hydrogen peroxide assay Enhancer each into a microcentrifuge tube containing the hydrogen peroxide assay buffer. This Fluorescent development solution is added to 20 µL of cell lysate, and each of the wells containing standard hydrogen peroxide assay and the solution is left to incubate in the dark for 10 minutes. The fluorescence is read at 530 nm/590 nm excitation and emission wavelength.

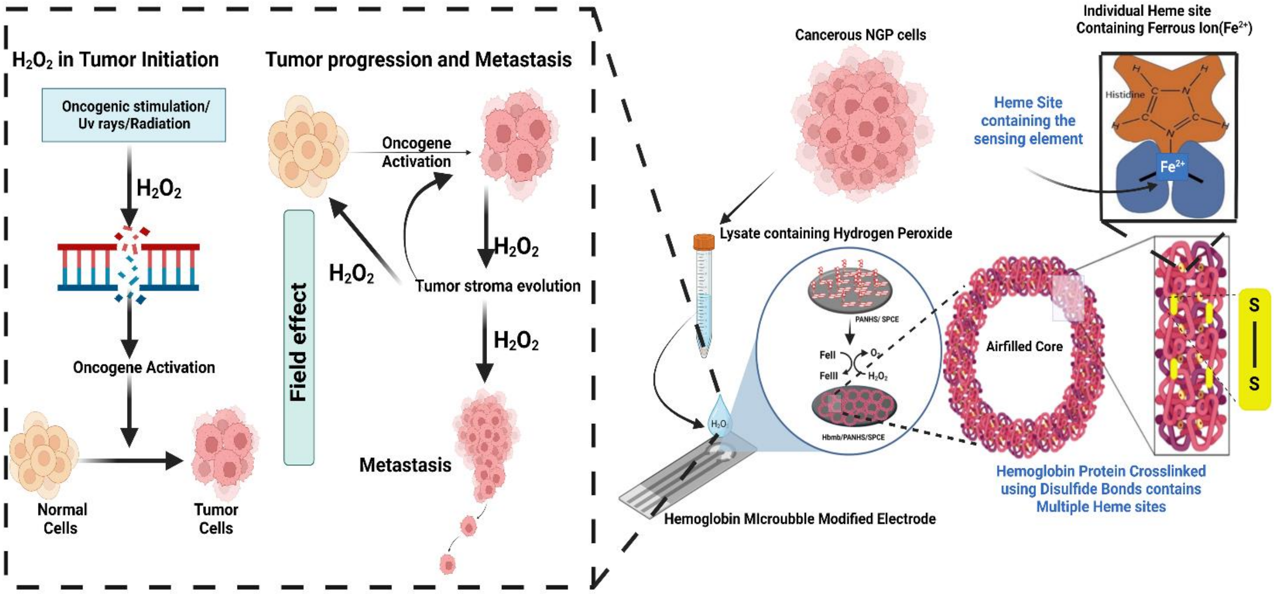

Figure S6. Shows the hemoglobin microbubble formed using inter-hemoglobin disulfide crosslinking. Each hemoglobin protein has four heme sites, microbubble created using crosslinked arrangement houses more heme sites than a single hemoglobin molecule. The schematic also includes the general pathophysiology of Hydrogen peroxide in tumor formation.

References

Elewi, A.S., Al-Shammaree, S.A.W., AL Sammarraie, A.K.M.A., 2020. Hydrogen peroxide biosensor based on hemoglobin-modified gold nanoparticles–screen printed carbon electrode. Sens. Bio-Sens. Res. 28, 100340. https://doi.org/10.1016/j.sbsr.2020.100340

Hu, Z., Dai, Z., Hu, X., Yang, B., Liu, Q., Gao, C., Zheng, X., Yu, Y., 2019. A facile preparation of FePt-loaded few-layer MoS2 nanosheets nanocomposites (F-MoS2-FePt NCs) and their application for colorimetric detection of H2O2 in living cells. J. Nanobiotechnology 17, 38. https://doi.org/10.1186/s12951-019-0465-3

Iwata, T., Mizutani, S., Okumura, K., Okumura, Y., Takahashi, K., Sawada, K., 2018. H2O2 Detection by Redox-based Potentiometric Sensors under Biological Environments. Sens. Mater. 30, 2359. https://doi.org/10.18494/SAM.2018.1947
