# Supplementary material for: H.O.S.T.: Hemoglobin microbubble-based Oxidative stress Sensing Technology
Source: Sci Rep. 2023 Sep 11;13:14942. doi: 10.1038/s41598-023-42050-z (PMC10495409; doi:10.1038/s41598-023-42050-z)
Supplement: Supplementary file 2 — Supplementary Information 2. [file 41598_2023_42050_MOESM2_ESM.pptx]

## Slide 1
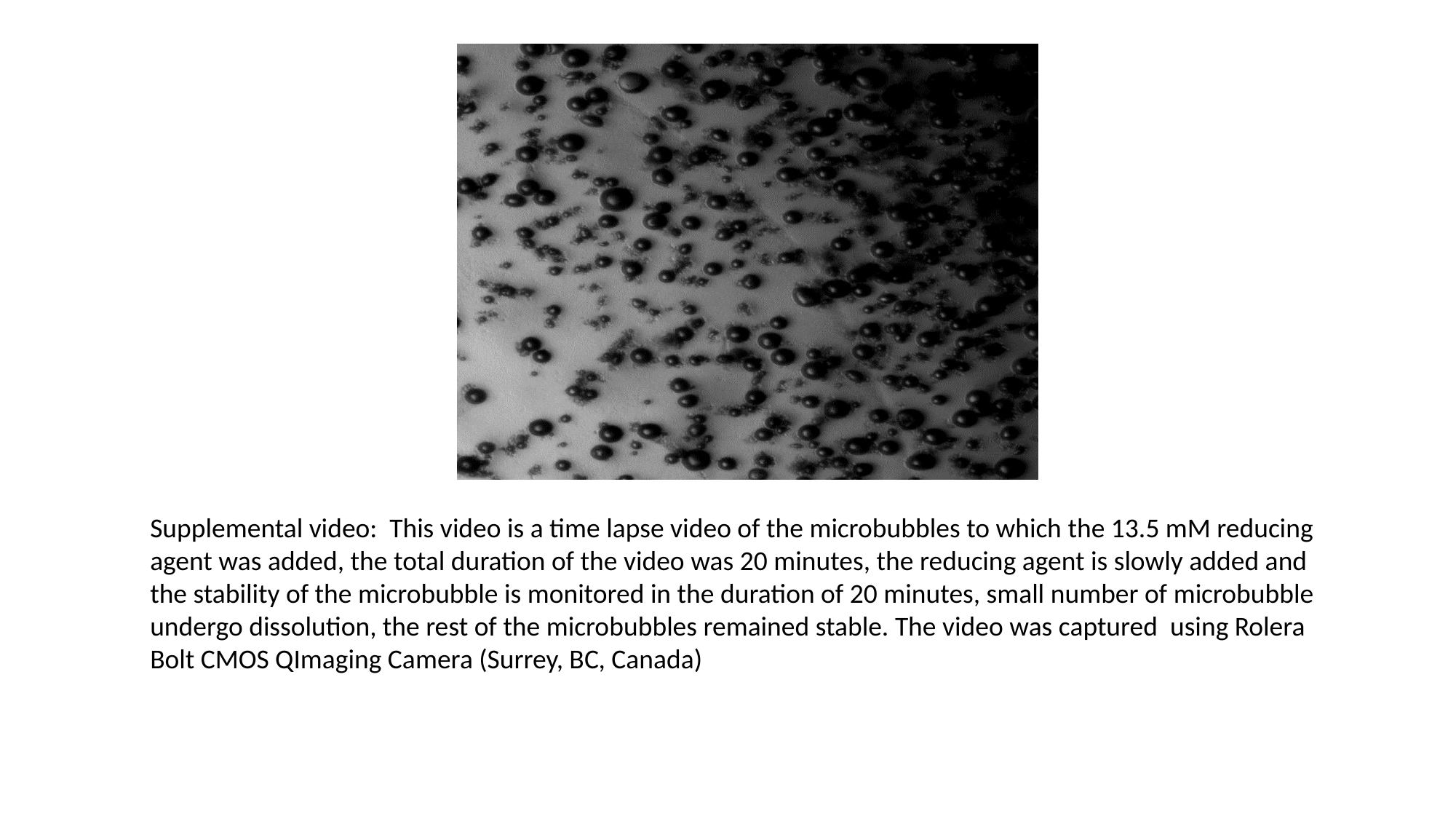

Supplemental video: This video is a time lapse video of the microbubbles to which the 13.5 mM reducing agent was added, the total duration of the video was 20 minutes, the reducing agent is slowly added and the stability of the microbubble is monitored in the duration of 20 minutes, small number of microbubble undergo dissolution, the rest of the microbubbles remained stable. The video was captured using Rolera Bolt CMOS QImaging Camera (Surrey, BC, Canada)
